# Comprehensive Virtual Screening of the Antiviral Potentialities of Marine Polycyclic Guanidine Alkaloids against SARS-CoV-2 (COVID-19)

**DOI:** 10.3390/biom11030460

**Published:** 2021-03-19

**Authors:** Amr El-Demerdash, Ahmed M. Metwaly, Afnan Hassan, Tarek Mohamed Abd El-Aziz, Eslam B. Elkaeed, Ibrahim H. Eissa, Reem K. Arafa, James D. Stockand

**Affiliations:** 1 Metabolic Biology & Biological Chemistry Department, John Innes Centre, Norwich Research Park, Norwich NR4 7UH, UK; 2 Organic Chemistry Division, Chemistry Department, Faculty of Science, Mansoura University, Mansoura 35516, Egypt; 3 Department of Pharmacognosy & Medicinal Plants, Faculty of Pharmacy (Boys), Al-Azhar University, Cairo 11884, Egypt; 4 Drug Design and Discovery Lab, Zewail City of Science and Technology, Giza 12578, Egypt; ahassan@zewailcity.edu.eg (A.H.); rkhidr@zewailcity.edu.eg (R.K.A.); 5 Biomedical Sciences Program, University of Science and Technology, Zewail City of Science and Technology, Giza 12578, Egypt; 6 Department of Cellular and Integrative Physiology, University of Texas Health Science Center at San Antonio, San Antonio, TX 78229-3900, USA; mohamedt1@uthscsa.edu; 7 Zoology Department, Faculty of Science, Minia University, El-Minia 61519, Egypt; 8 Department of Pharmaceutical Sciences, College of Pharmacy, AlMaarefa University, Ad Diriyah 13713, Riyadh, Saudi Arabia; ikaeed@mcst.edu.sa; 9 Pharmaceutical Medicinal Chemistry & Drug Design Department, Faculty of Pharmacy (Boys), Al-Azhar University, Cairo 11884, Egypt; ibrahimeissa@azhar.edu.eg

**Keywords:** virtual screening, docking, COVID-19, antiviral, cytotoxicity, guanidine alkaloids, crambescidins, crambescins, *Monanchora* n. sp.

## Abstract

The huge global expansion of the COVID-19 pandemic caused by the novel SARS-corona virus-2 is an extraordinary public health emergency. The unavailability of specific treatment against SARS-CoV-2 infection necessitates the focus of all scientists in this direction. The reported antiviral activities of guanidine alkaloids encouraged us to run a comprehensive in silico binding affinity of fifteen guanidine alkaloids against five different proteins of SARS-CoV-2, which we investigated. The investigated proteins are COVID-19 main protease (M^pro^) (PDB ID: 6lu7), spike glycoprotein (PDB ID: 6VYB), nucleocapsid phosphoprotein (PDB ID: 6VYO), membrane glycoprotein (PDB ID: 6M17), and a non-structural protein (nsp10) (PDB ID: 6W4H). The binding energies for all tested compounds indicated promising binding affinities. A noticeable superiority for the pentacyclic alkaloids particularly, crambescidin 786 (**5**) and crambescidin 826 (**13**) has been observed. Compound **5** exhibited very good binding affinities against Mpro (ΔG = −8.05 kcal/mol), nucleocapsid phosphoprotein (ΔG = −6.49 kcal/mol), and nsp10 (ΔG = −9.06 kcal/mol). Compound **13** showed promising binding affinities against M^pro^ (ΔG = −7.99 kcal/mol), spike glycoproteins (ΔG = −6.95 kcal/mol), and nucleocapsid phosphoprotein (ΔG = −8.01 kcal/mol). Such promising activities might be attributed to the long ω-fatty acid chain, which may play a vital role in binding within the active sites. The correlation of c Log P with free binding energies has been calculated. Furthermore, the SAR of the active compounds has been clarified. The Absorption, Distribution, Metabolism, Excretion, and Toxicity (ADMET) studies were carried out in silico for the 15 compounds; most examined compounds showed optimal to good range levels of ADMET aqueous solubility, intestinal absorption and being unable to pass blood brain barrier (BBB), non-inhibitors of CYP2D6, non-hepatotoxic, and bind plasma protein with a percentage less than 90%. The toxicity of the tested compounds was screened in silico against five models (FDA rodent carcinogenicity, carcinogenic potency TD_50_, rat maximum tolerated dose, rat oral LD_50_, and rat chronic lowest observed adverse effect level (LOAEL)). All compounds showed expected low toxicity against the tested models. Molecular dynamic (MD) simulations were also carried out to confirm the stable binding interactions of the most promising compounds, **5** and **13,** with their targets. In conclusion, the examined 15 alkaloids specially **5** and **13** showed promising docking, ADMET, toxicity and MD results which open the door for further investigations for them against SARS-CoV-2.

## 1. Introduction

COVID-19 is a disease caused by a new strain of the coronavirus. This disease first appeared in Wuhan, China at the end of December 2019. Two months later, the disease became widespread in China [1,2]. COVID-19 has now turned into a pandemic affecting almost every country in the world. As of 1 December 2020, COVID-19 has affected more than 63,697,245 patients in more than 188 countries and territories around the world and caused around 1,477,645 deaths worldwide. Unfortunately, there are no specific antiviral medications available for the treatment of COVID-19 patients. Many scientists worldwide are working to prepare a vaccine to fight COVID-19 infection. At present, several vaccines have been approved for clinical trials at home and abroad.

**Figure 1:**
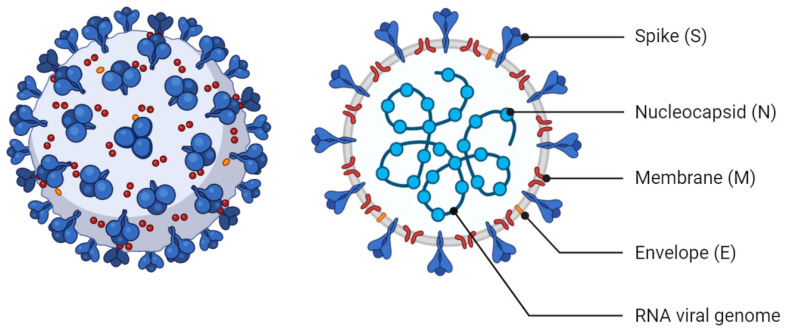
Schematic representation of the structure of SARS-CoV-2. It has at least four canonical structural proteins; E (envelope), M (membrane), N (nucleocapsid) and S (spike) proteins (Created with BioRender.com).

Coronavirus viruses belong to the order Nidovirales in the subfamily Coronavirinae (family Coronaviridae) [3]. They are enveloped viruses that contain a large non-segmented, positive-sense RNA genome with a length of up to 33.5 kilobases [4]. The Coronaviridae family can be classified into four genera to include Alpha-, Beta-, Gamma- and Delta-coronavirus (alphaCoV, betaCoV, gammaCoV, and deltaCoV). Coronaviruses were named for how they appear under the electron microscope. The viruses look like they are covered with pointed structures that surround them like a corona or crown due to the presence of spike glycoproteins on their envelope (Figure 1) [5].

Coronaviruses mostly cause insignificant respiratory infections, including the common cold. However, more recent emerging coronaviruses can cause more serious diseases, including severe acute respiratory syndrome (SARS-CoV) and the Middle East respiratory syndrome (MERS-CoV) [6,7]. SARS-CoV was first detected in 2002 in Foshan, China, possibly originated from the Chinese horseshoe bat-CoV, 35 to 20 years ago via zootonic transmission from the civet [8,9,10,11]. MERS-CoV detected in 2012 in the Arabian Peninsula, possibly originated from the South African Bat-CoV, around 14 years ago via zootonic transmission from the camel [8,9,12]. SARS-CoV-2 detected in 2019 in Wuhan, China, possibly originated from intermediate horseshoe bat-CoV around 11 years ago via zootonic transmission from pangolins [13,14,15].

Generally, viral proteins can be classified according to their functions into two major groups as structural and non-structural proteins [16]. Structural proteins, such as nucleocapsid proteins, can function as shields protecting viral DNA from being degraded by host enzymes [17]. Other vital structural proteins are the membrane glycoproteins which form an envelope enclosing the virus capsid and bind to specific receptors on host cell membranes [18]. For example, the coronavirus spike glycoprotein (S protein), by binding to a specific cellular receptor, is a significant structural protein that mediates entry into cells [19]. The main protease (Mpro) is a key non-structural chymotrypsin-like cysteine proteases enzyme used by coronaviruses for replication. It acts on the two large polyproteins (PPs) (PP1a and PP1ab) to release the 16 non-structural proteins (NSPs 1–16) through cleavage of the C-terminal end of these PPs [20,21]. The non-structural protein (nsp10) by functioning as a vital cofactor is a crucial regulator of the replicative enzyme SARS-CoV replicas [22].

Given the fact that oceans and seas cover almost 70% of the earth, and consequently, contain the largest ecological diversity of biological species, marine natural products (MNPs) attract much interest. This includes metabolite congers from the marine sponge *Cryptotethya crypta* [23]. MNPs, many of which have distinct structures and biological mechanisms, represent a huge renewable natural reservoir for possible new drugs [24,25,26,27,28,29,30,31,32,33,34,35]. Among the eight clinically approved marine drugs, two successful molecules were identified as antiviral drugs, namely cytarabine (Cytosar-U, Depocyt) and vidarabine (Vira-A). These are synthetic analogues originally inspired by spongothymidine, which is the first nucleoside isolated from the sponge *Cryptotethya crypta*. Both compounds hinder viral DNA polymerase and consequently, DNA synthesis in particular herpes simplex virus type 1 and type 2, vaccinia and varicella zoster viruses [26]. Additionally, two marine-derived molecules are being preclinically investigated for their antiviral-HIV-1, HIV-2, and SIV activities. These are avarol, a sesquiterpenoid hydroquinone isolated from the marine sponge *Dysidea avara*, and cyanovirin-N, a protein isolated from cultures of the cyanobacterium (blue-green alga) *Nostoc ellipsosporum* [36]. Meanwhile, recent synthetic efforts and clinical trials highlight the exploration of an additional 19 structurally divergent MNP, many of which are nucleosides, as antivirals [37].

Polycyclic guanidine alkaloids (PGAs) represent a major group of marine metabolites common to Poecilosclerida sponges including *Batzella*, *Crambe*, *Monanchora*, *Clathria*, *Ptilocaulis*, and some starfishes, such as *Fromia monilis* and *Celerina heffernani* [38,39,40]. Since the discovery of the first antiviral pentacyclic congener, ptilomycalin A, in 1989 by Kashman and coworkers [41], these metabolites have attracted much interest. Chemically, PGAs contain a common central tricyclic guanidinic core (Vessel) linked to a ω-long chain fatty acid (Anchor). They are synthesized via the aza-Michael incorporation of a polyketide chain with a guanidinic moiety, followed by subsequent cyclizations, substitutions, and oxidations. These chemical reactions produce a structurally complex and diverse group of molecules that have a central guanidinic core, including bicyclic (e.g., crambescins), tricyclic (e.g., batzelladines) and pentacyclic (e.g., crambescidins) derivatives [38,39,40,41,42]. PGA metabolites are recognized for displaying a broad spectrum of biomedical properties, including being cytotoxic [43,44,45,46,47,48,49,50], antimicrobial [51,52], antifungal [53,54], antimalarial, and anti-infective [55,56,57,58]; as well as being enzyme inhibitors and Ca+2 channel blockers [59,60]. Moreover, many PGAs have been reported to display significant antiviral activities against HIV-1, herpes simplex type-1 [41,43,61,62,63,64,65,66,67]. Indeed, polycyclic guanidinic meltabilities including tricyclic batzelladines and pentacyclic crambescidins isolated from the marine sponges *Crambe crambe* and *Monanchora unguifera* and their synthetic analogues displayed significant inhibitory activity against gp120-CD4 binding, motivate CD4-p56lck dissociation, and prevent HIV-1 cell fusion [68,69,70,71].

As part of our research into MNPs together with the global effort to find new robust antiviral drugs capable of combating COVID-19, we report here on the potential interactions between five SARS-CoV-2 proteins and fifteen structurally diverse polycyclic guanidine-containing alkaloids isolated from the Pacific marine sponge *Monanchora* n. sp. [45].

## 2. Materials and Methods 

### 2.1. Docking Studies

The crystal structures of the target proteins: (i) COVID-19 main protease (M^pro^) (PDB ID: 6lu7, resolution: 2.16 Å), (ii) spike glycoproteins (PDB ID: 6VYB, resolution: 3.20 Å), (iii) nucleocapsid phosphoprotein (PDB ID: 6VYO, resolution: 1.70 Å), (iv) membrane glycoprotein (PDB ID: 6M17, resolution: 2.90 Å), and (v) nsp10 (PDB ID: 6W4H, resolution: 1.80 Å) were downloaded from Protein Data Bank (http://www.pdb.org). Molecular Operating Environment (MOE) was used for the docking analysis [72]. In these studies, the free energies and binding modes of the examined molecules against target proteins were determined. At first, the water molecules were removed from the crystal structures of target proteins, retaining only main-chain amino acids which are essential for binding. The co-crystallized ligands were used as reference ligands. Then, the protein structures were protonated, and the hydrogen atoms were hidden. Next, the energy was minimized and the binding pockets of each protein were defined [73,74]. The structures of the examined compounds and the co-crystallized ligands were drawn using ChemBioDraw Ultra 14.0 and saved using SDF formats. Then, the saved files were opened using MOE software and 3D structures were protonated. Next, the energy of the molecules was minimized. Validation processes were performed for each target receptor by running the docking process only for the co-crystallized ligand. Low Root Mean Square Deviation (RMSD) values between docked and crystal conformations indicated valid performances [75,76]. The docking procedures were carried out utilizing a default protocol. In each case, 10 docked structures were generated using genetic algorithm searches. The output from MOE software was further analyzed and visualized using Discovery Studio 4.0 software [76,77,78,79].

### 2.2. ADMET

ADMET descriptors (Absorption, Distribution, Metabolism, Excretion, and Toxicity) of the compounds were determined using Discovery Studio 4.0. Initially, the Chemistry at Harvard Macromolecular Mechanics (CHARMM) force field was applied, then the compounds were prepared and minimized according to the preparation for small molecules protocol. Then ADMET descriptors protocol was applied to carry out these studies [75,78].

### 2.3. Toxicity

The toxicity parameters were calculated using Discovery Studio 4.0. Daclatasvir was used as a reference drug. Initially, the CHARMM force field was applied, then the compounds were prepared and minimized according to the preparation for small molecules protocol. Then different parameters were calculated using toxicity prediction (extensible) protocols.

### 2.4. Isolation and Characterization of Compounds ***1**–**15***

Compounds **1**–**15** were isolated and identified from the French Polynesian marine sponge, *Monanchora* n. sp. For detailed isolation and structural characterizations, see El-Demerdash et al. [45].

### 2.5. Molecular Dynamics Simulation for Compounds ***5*** and ***13***

Since compound **5** (crambescidins 786) and compound **13** (crambescidins 826) displayed the best binding modes and free energies among the 15 investigated compounds, they were subjected to molecular dynamics (MD) investigation. A 100 ns MD simulation was performed on compound **5** binding against the COVID-19 main protease, nucleocapsid phosphoprotein and nsp10. Furthermore, a 100 ns MD simulation was performed on compound **13** bounded to the COVID-19 main protease, spike glycoproteins, and nucleocapsid phosphoprotein.

MD simulations were performed using GROMACS 2019 software package where the CHARMM36 forcefield was used for protein topology preparation and the official CHARMM General Force Field server (CGenFF) for ligand topology preparation solvated in a dodecahedron box of common simple point charge (SPC) water model applying explicit-solvent periodic boundary conditions. The 6 solvated complexes were neutralized using sodium and chloride ions. Both systems were subjected to minimization using the steepest descent method through 5000 steps to resolve any steric clashes or inappropriate geometry. Then, to ensure a reasonable starting structure, the system was equilibrated under constant number of particles, volume, and temperature (NVT) ensemble for 100 ps using a Berendsen thermostat. The second round of equilibration was performed under constant pressure (Isothermal-isobaric (NPT) ensemble) using the Parrinello–Rahman barostat for an additional 100 ps. Finally, the position restraints were released, and the system was simulated in the production run under an NPT ensemble (Nosé–Hoover thermostat and Parrinello–Rahman barostat) for 100 ns using a time step of 2 fs. Simulation results were analyzed using Visual Molecular Dynamics (VMD) software, ver.1.9.3 [80].

## 3. Results

**Chart 1:**
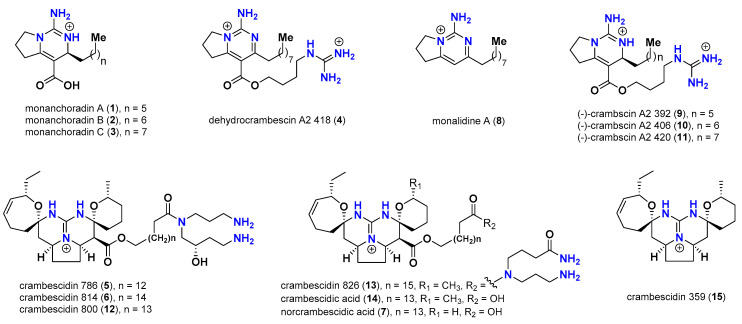
Reported polycyclic guanidine alkaloids (**1**–**15**) from *Monanchora* n. sp. marine sponge.

In this work, the binding potential of 15 guanidine-containing marine alkaloids (**1**–**15**), previously isolated from the French Polynesian *Monanchora* n. sp. marine sponge (Chart 1), against a host of SARS-CoV-2 proteins has been investigated. Five SARS-CoV-2 proteins (structural and non-structural) were selected. These include: (i) the COVID-19 main protease (M^pro^) (PDB ID: 6lu7, resolution: 2.16 Å), (ii) the spike glycoproteins (PDB ID: 6VYB, resolution: 3.20 Å), (iii) the nucleocapsid phosphoprotein (PDB ID: 6VYO, resolution: 1.70 Å), (iv) the membrane glycoprotein (PDB ID: 6M17, resolution: 2.90 Å), and (v) the nonstructural protein (nsp10) (PDB ID: 6W4H, resolution: 1.80 Å). Comprehensive docking studies were performed using MOE 14.0 software. These docking studies predicted the free energy (ΔG) of binding specifically for the molecules shown in Table 1.

### 3.1. Validation of the Docking Processes 

**Figure 2:**
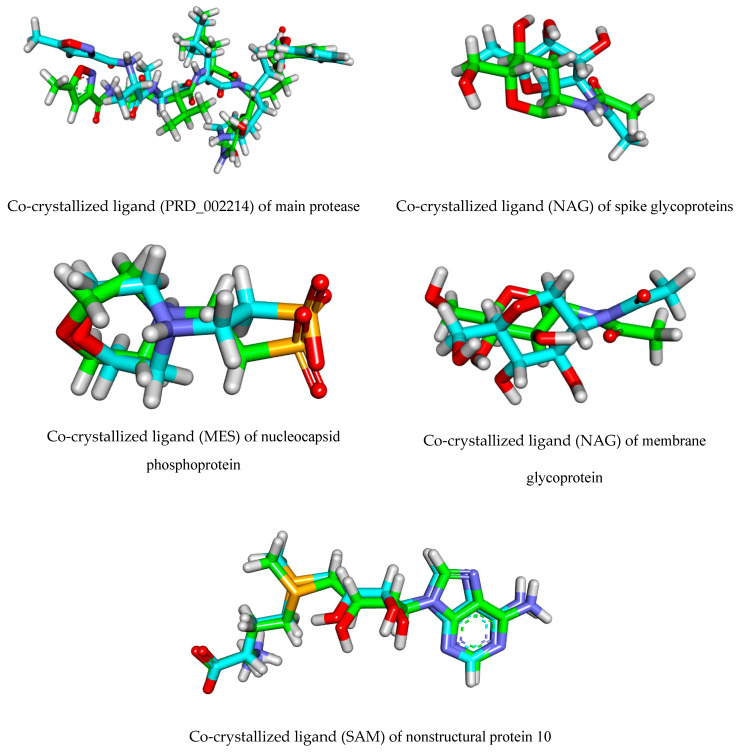
Superimposition of the co-crystallized poses (green) and the docking poses (maroon) of the same ligands.

Validation of the docking procedure was achieved via redocking of the co-crystallized ligands against the active pockets of SARS-CoV-2 target proteins. The calculated RMSD values between the redocked poses and the co-crystallized ligands were 2.6, 2.1, 0.9, 2.4, and 0.5 for COVID-19 main protease, spike glycoproteins, nucleocapsid phosphoprotein, membrane glycoprotein, and nonstructural protein 10, respectively. Such values of RMSD indicated the efficiency and validity of the docking processes (Figure 2).

Docking studies, in general, showed robust binding energies for all compounds tested with a noticeable superiority for pentacyclic compounds. The pentacyclic guanidines, crambescidins 786 (**5**) and 826 (**13**) exhibited the greatest free energy of docking. Crambescidin 786 (**5**) showed promising binding affinities against COVID-19 main protease (ΔG = −8.05 kcal/mol), nucleocapsid phosphoprotein (ΔG = −6.49 kcal/mol), and nsp10 (ΔG = −9.06 kcal/mol), compared to the co-crystallized ligands PRD_002214 (ΔG = −8.18 kcal/mol), MES (ΔG = −3.80 kcal/mol), and SAM (ΔG = −5.77 kcal/mol), respectively. In addition, crambescidin 826 (**13**) showed good binding affinities against the COVID-19 main protease (ΔG = −7.99 kcal/mol), spike glycoproteins (ΔG = −6.95 kcal/mol), and nucleocapsid phosphoprotein (ΔG = −8.01 kcal/mol), compared to the co-crystallized ligands PRD_002214 (ΔG = −8.18 kcal/mol), NAG (ΔG = −3.56 kcal/mol), and MES (ΔG = −3.80 kcal/mol), respectively (Table 1).

**Figure 3:**
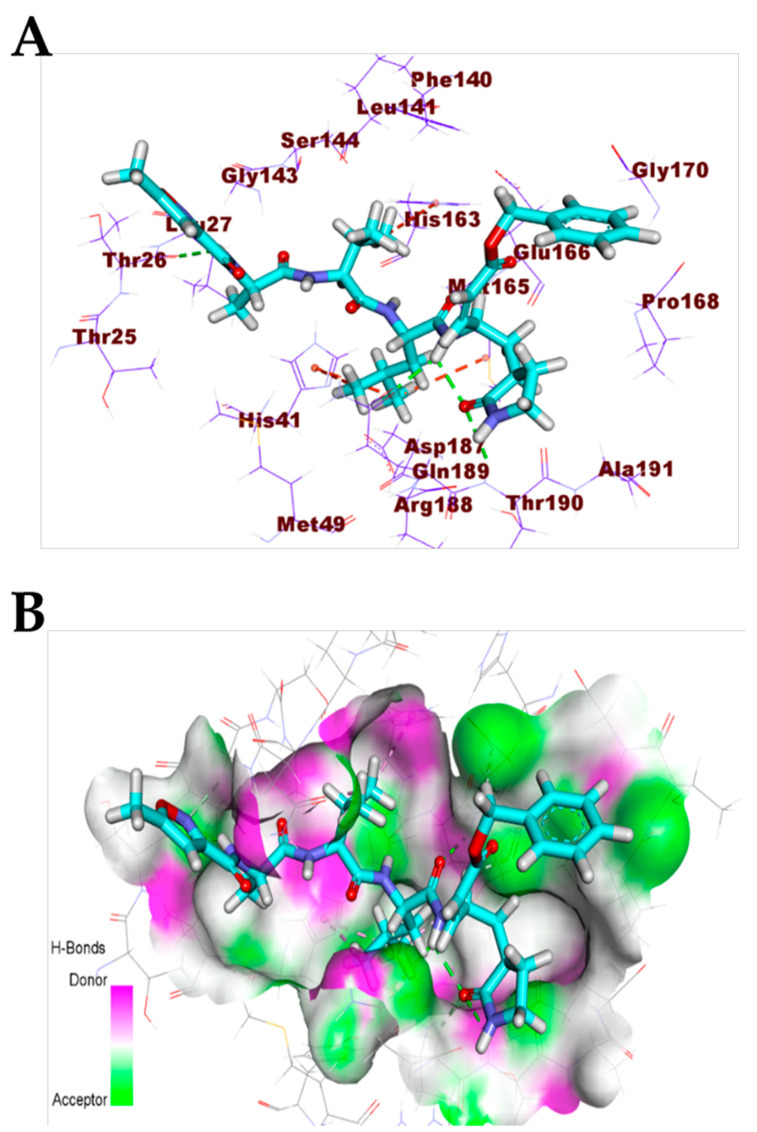
(**A**) Co-crystallized ligand (PRD_002214) docked into the active site of COVID-19 main protease. (**B**) Mapping surface showing co-crystallized ligand (PRD_002214) occupying the active pocket of COVID-19 main protease.

**Figure 4:**
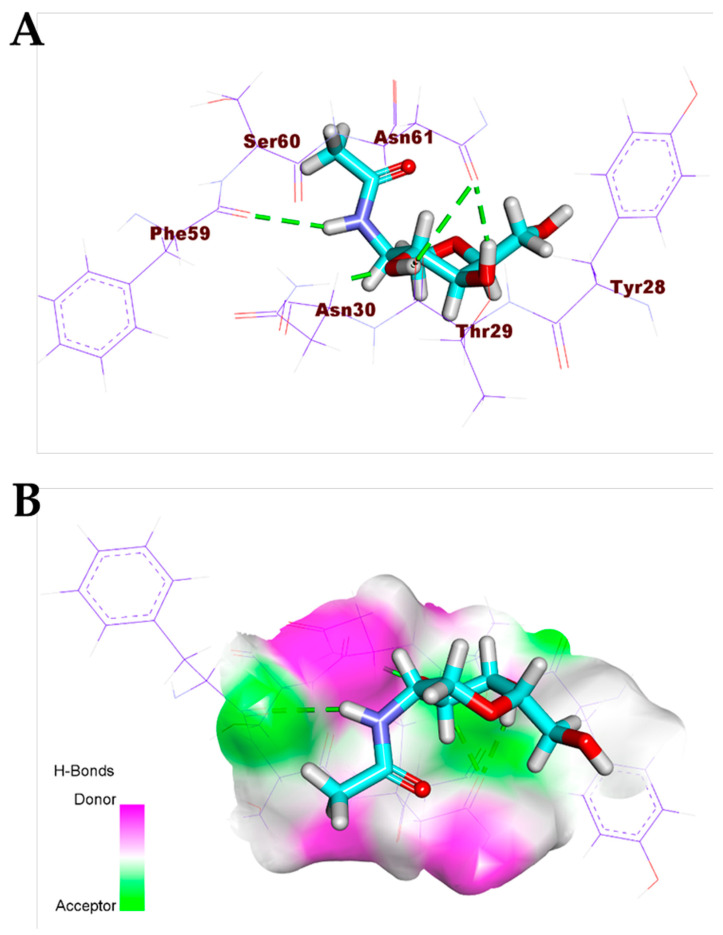
(**A**) Co-crystallized ligand (NAG) docked into the active site of COVID-19 spike glycoprotein. (**B**) Mapping surface showing co-crystallized ligand (NAG) occupying the active pocket of COVID-19 spike glycoproteins.

The detailed binding mode of the co-crystallized ligand (PRD_002214) against COVID-19 main protease was as follows: the ligand formed four hydrogen bonds and three hydrophobic interactions. In addition, the 2-oxopyrrolidin-3-yl moiety occupied the first pocket of (M^pro^) and the isopropyl moiety occupied the second pocket of (M^pro^). Furthermore, the benzyl acetate moiety occupied the third pocket of the receptor. Moreover, the 5-methylisoxazole-3-carboxamide moiety was incorporated in the fourth pocket (Figure 3). For the binding mode of the co-crystallized ligand (NAG) against COVID-19 spike glycoprotein, it formed five hydrogen bonds with Asn61, Asn30, The29, and Phe59 (Figure 4).

**Figure 5:**
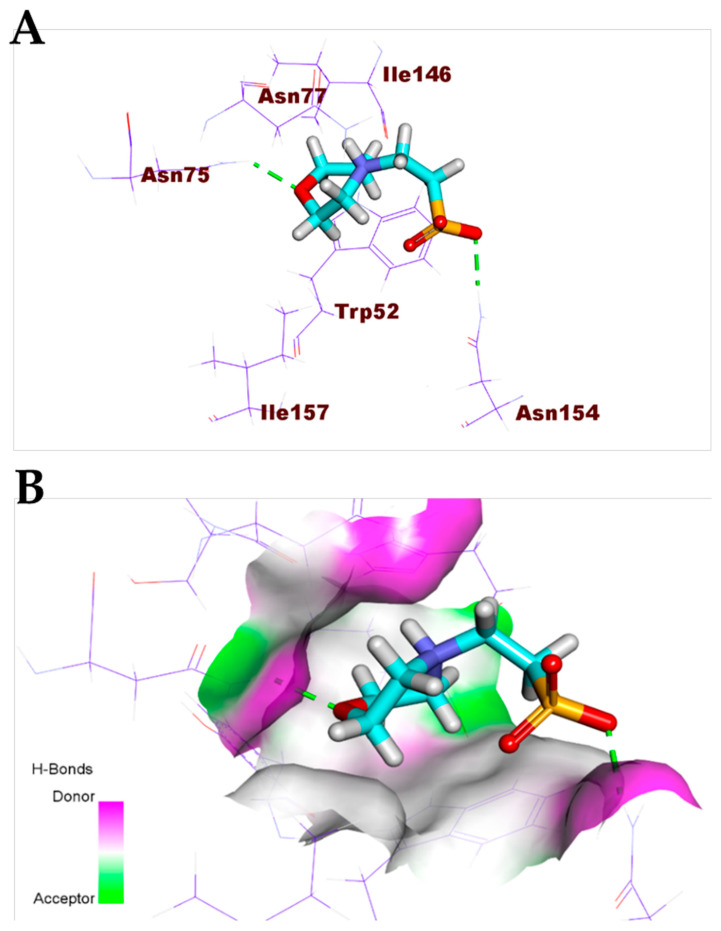
(**A**) Co-crystallized ligand (MES) docked into the active site of COVID-19 nucleocapsid phosphoprotein. (**B**) Mapping surface showing co-crystallized ligand (MES) occupying the active pocket of COVID-19 nucleocapsid phosphoprotein.

**Figure 6:**
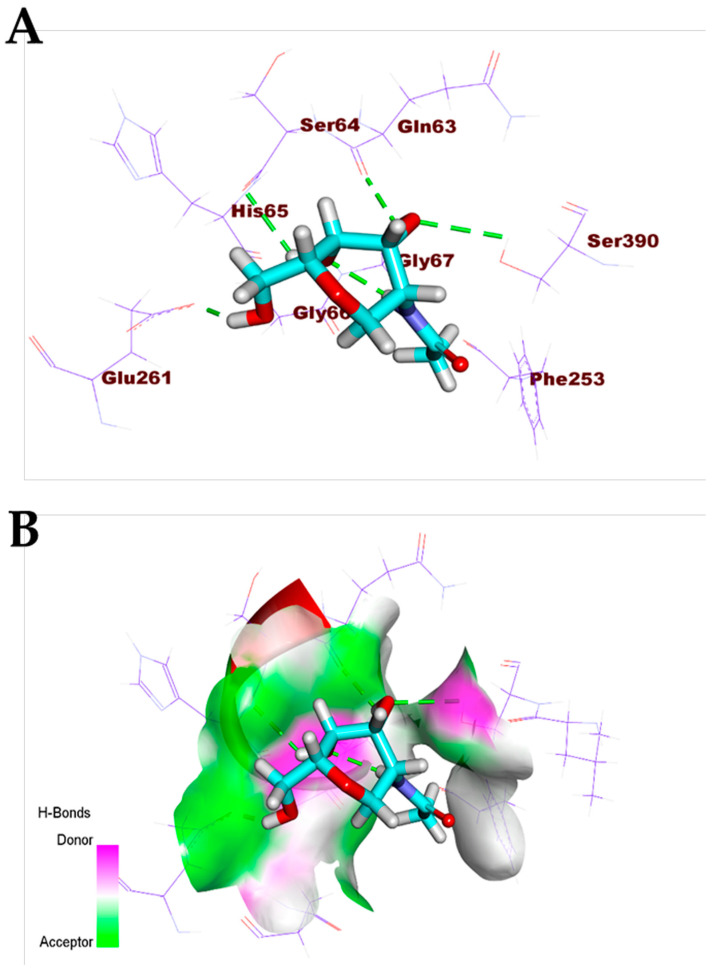
(**A**) Co-crystallized ligand (NAG)docked into the active site of COVID-19 membrane glycoprotein. (**B**) Mapping surface showing co-crystallized ligand (NAG) occupying the active pocket of COVID-19 membrane glycoprotein.

**Figure 7:**
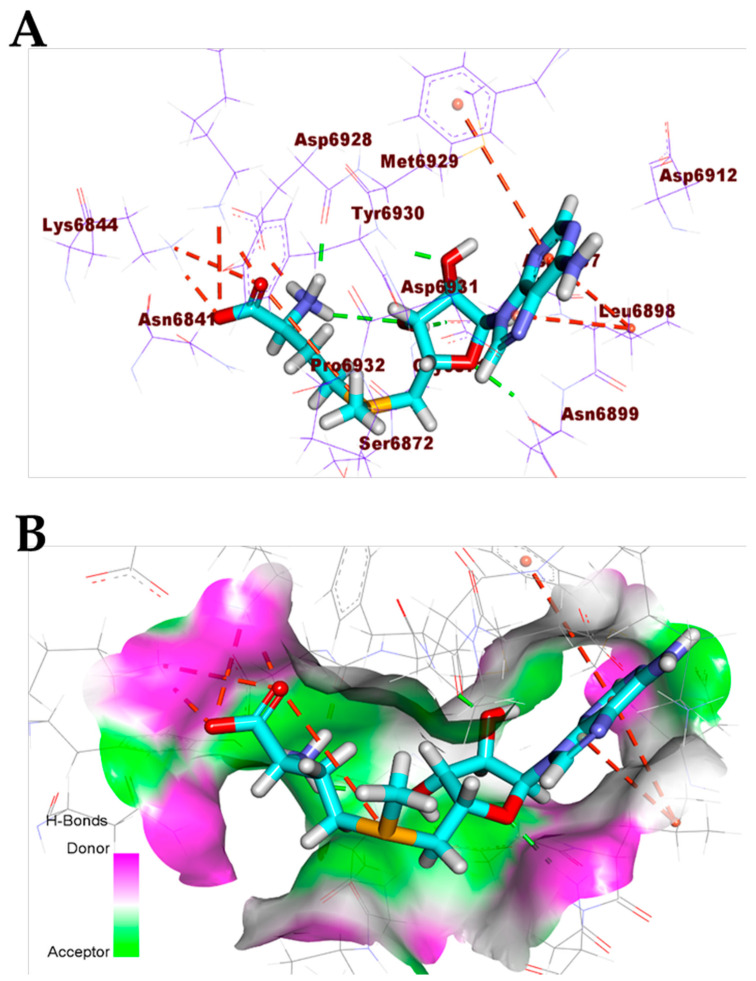
(**A**) Co-crystallized ligand (SAM) docked into the active site of COVID-19 nsp10. (**B**) Mapping surface showing co-crystallized ligand (SAM) occupying the active pocket of COVID-19 nsp10.

Additionally, the co-crystallized ligand (MES) bonded with COVID-19 nucleocapsid phosphoprotein through the formation of two hydrogen bonds with Asn154 and Asn75 (Figure 5). Furthermore, the co-crystallized ligand (NAG) docked into the active site of COVID-19 membrane glycoprotein showed four hydrogen bonds with Ser390, Ser64, Glu261, and Gln63 (Figure 6). Finally, the binding mode of the co-crystallized ligand (SAM) against COVID-19 nsp10 showed three hydrogen bonds with Asn6899, Tyr6930, Asp6928, and Asp6897. Moreover, it formed seven hydrophobic interactions with Lys6968, Lys6844, Asp6928, Phe6947, and Leu6898 (Figure 7).

**Figure 8:**
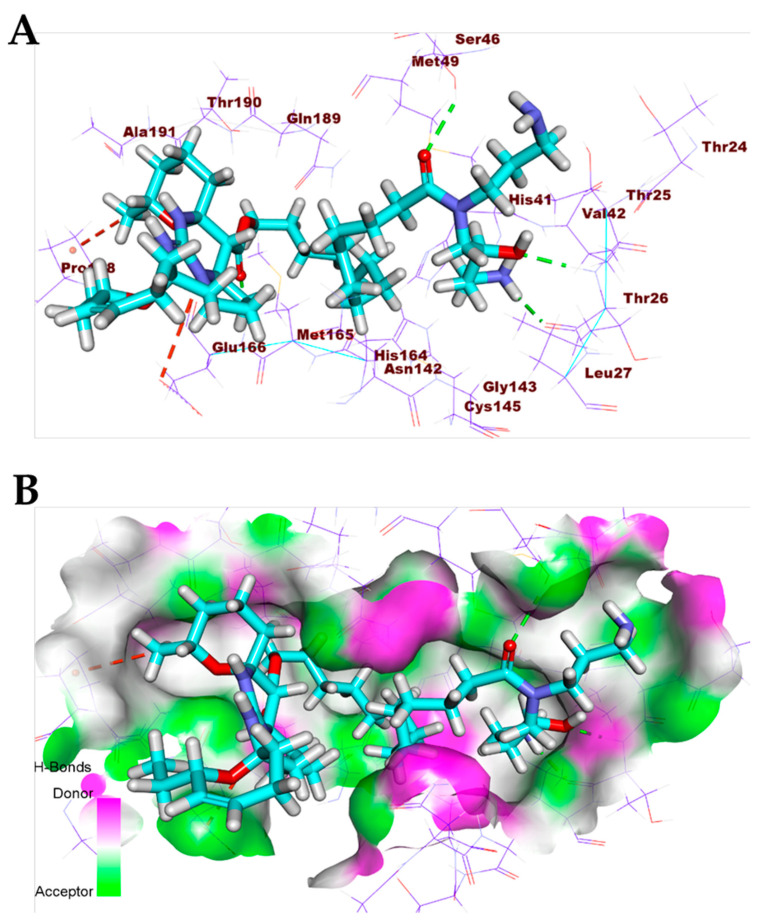
(**A**) Compound **5** docked into the active site of COVID-19 main protease. (**B**) Mapping surface showing compound **5** occupying the active pocket of COVID-19 main protease.

**Figure 9:**
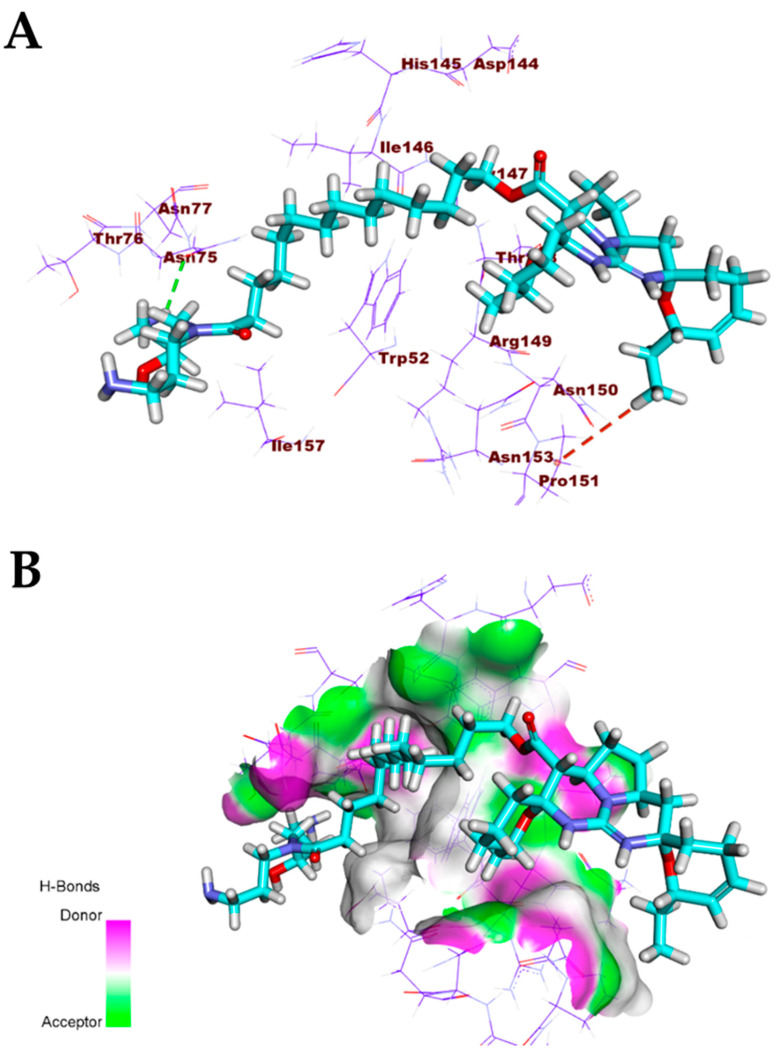
(**A**) Compound **5** docked into the active site of COVID-19 nucleocapsid phosphoprotein. (**B**) Mapping surface showing compound **5** occupying the active pocket of COVID-19 nucleocapsid phosphoprotein.

**Figure 10:**
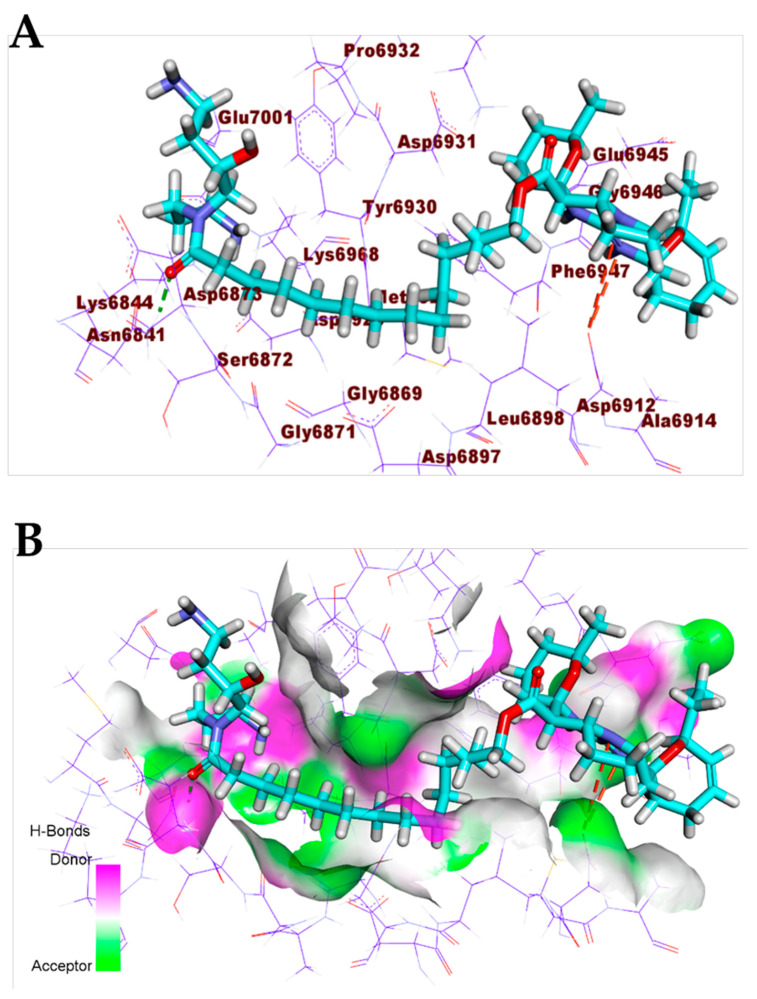
(**A**) Compound **5** docked into the active site of COVID-19 nsp10. (**B**) Mapping surface showing compound **5** occupying the active pocket of COVID-19 nsp10.

The pentacyclic crambescidin 786 (**5**) exhibited a binding mode similar to that of the co-crystallized ligands against the COVID-19 main protease, nucleocapsid phosphoprotein, and nsp10. The binding mode of compound **5** against the COVID-19 main protease showed four hydrogen bonds with Thr26, Ser46, and Glu166. In addition, it formed two hydrophobic interactions with Lul166 and Pro168. The long ω-fatty acid chain facilitated the occupation of compound **5** with different pockets of the (M^pro)^ (Figure 8). For the binding mode of **5** against the COVID-19 nucleocapsid phosphoprotein, it occupied the binding region of the target protein forming one hydrogen bond with Asn75 and one hydrophobic interaction with Pro151 (Figure 9). Finally, the binding mode of **5** against COVID-19 nsp10 showed one hydrogen bond with Asn6841 and two electrostatic interactions with Asp6912. The ω-fatty acid chain of compound **5** played a vital role in the occupancy of the active site of the target protein (Figure 10).

**Figure 11:**
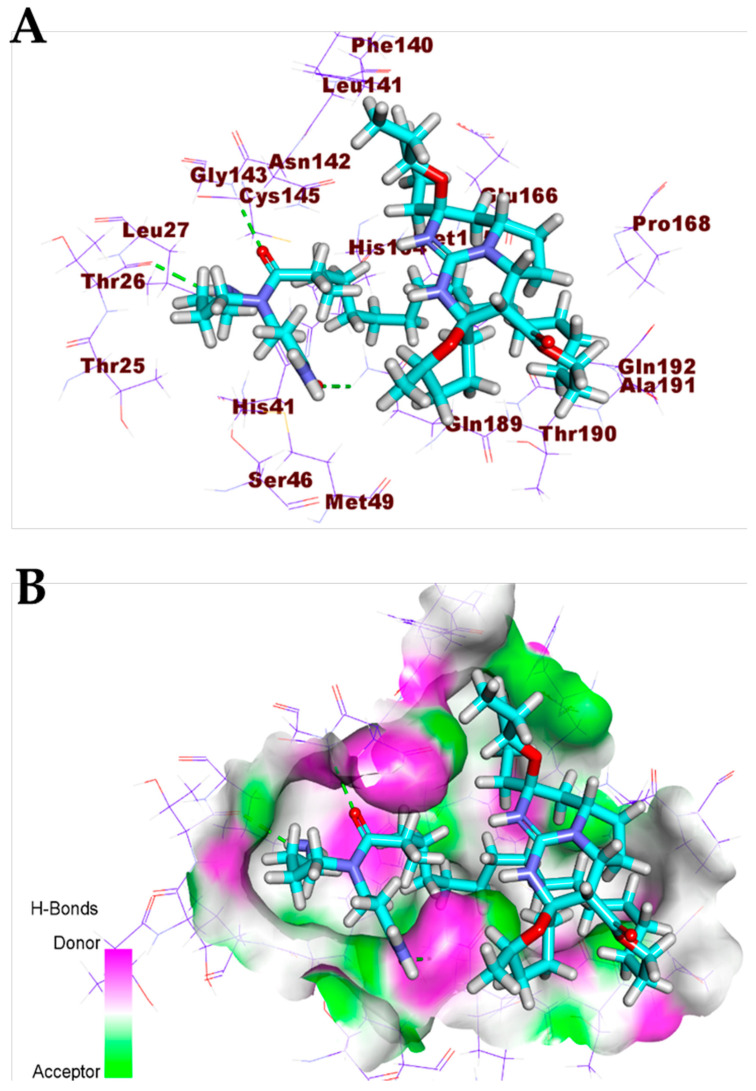
(**A**) Compound **13** docked into the active site of COVID-19 main protease. (**B**) Mapping surface showing compound **13** occupying the active pocket of COVID-19 main protease.

**Figure 12:**
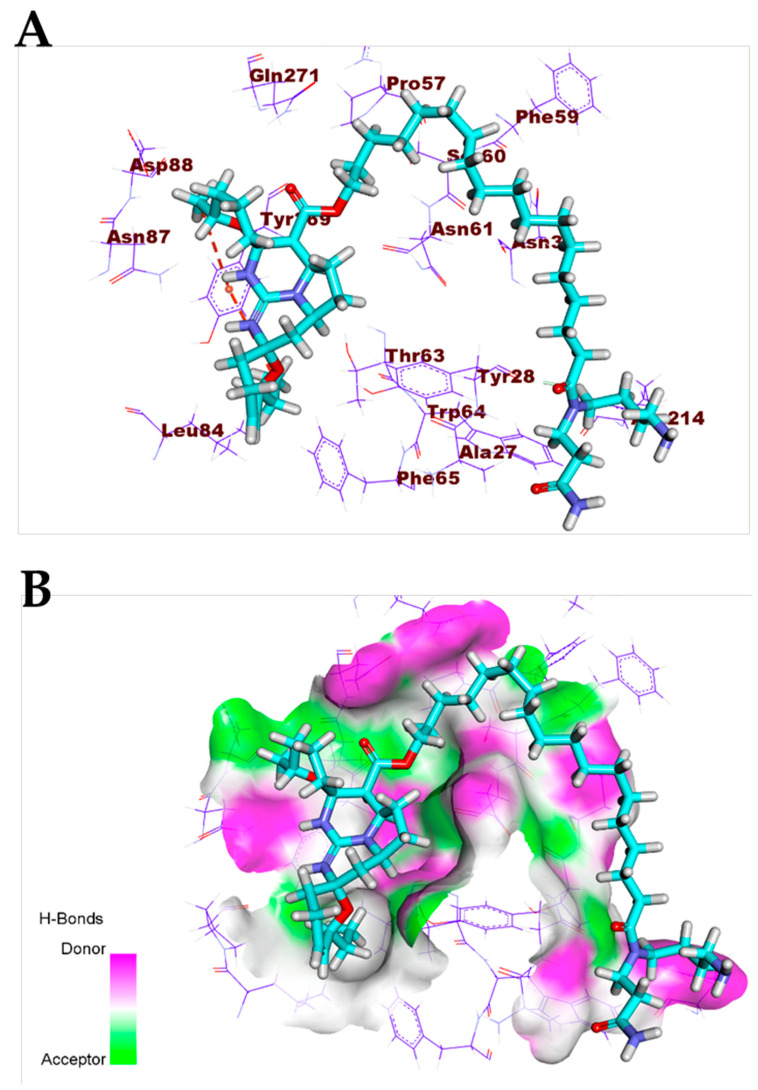
(**A**) Compound **13** docked into the active site of COVID-19 spike glycoprotein. (**B**) Mapping surface showing compound **13** occupying the active pocket of COVID-19 spike glycoproteins.

**Figure 13:**
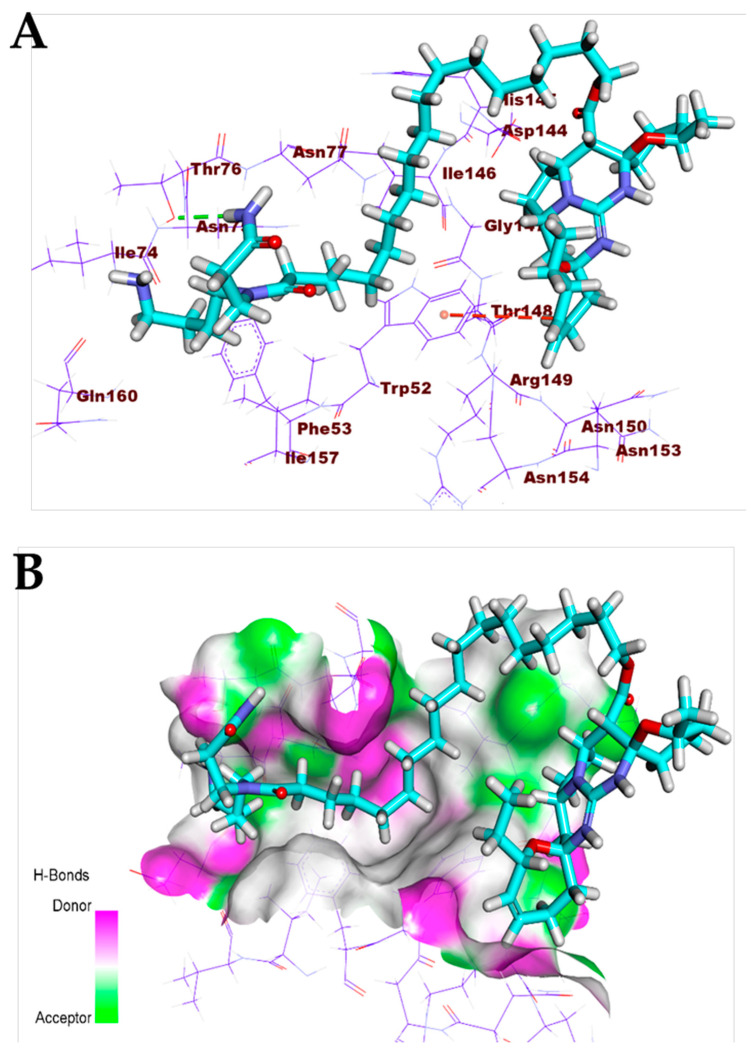
(**A**) Compound **13** docked into the active site of COVID-19 nucleocapsid phosphoprotein. (**B**) Mapping surface showing compound **13** occupying the active pocket of COVID-19 nucleocapsid phosphoprotein.

**Figure 14:**
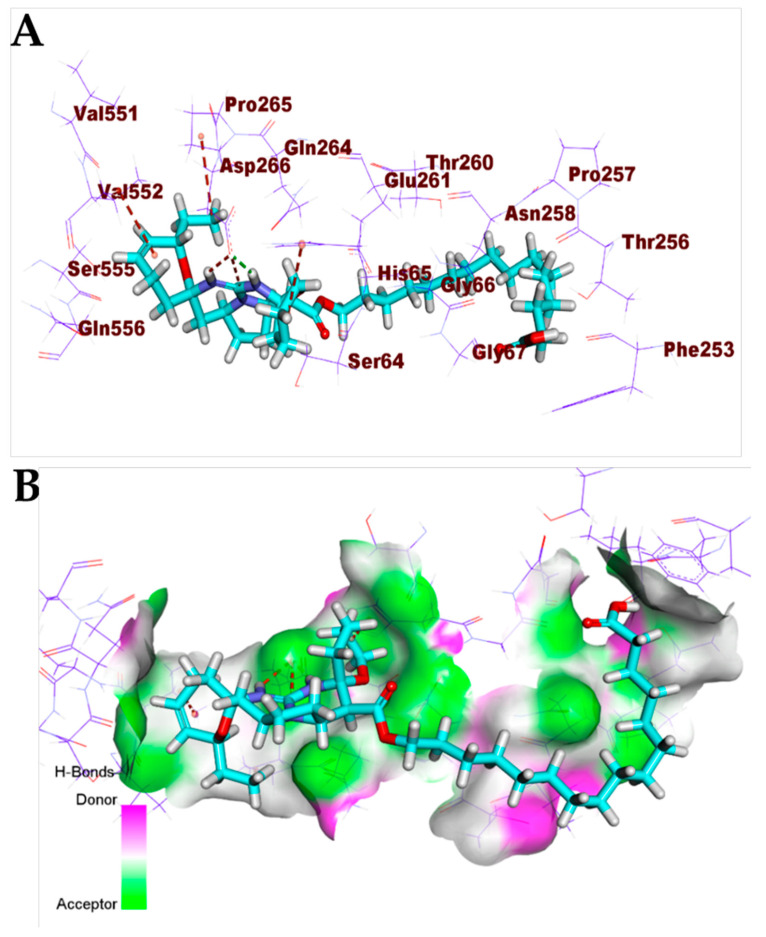
(**A**) Compound **7** docked into the active site of COVID-19 membrane glycoprotein. (**B**) Mapping surface showing compound **7** occupying the active pocket of COVID-19 membrane glycoprotein.

Crambescidin 826 (**13**) exhibited a binding mode like that of the co-crystallized ligands against the COVID-19 main protease, spike glycoproteins, and nucleocapsid phosphoprotein. The binding mode of compound **13** against the COVID-19 main protease showed three hydrogen bonds with Gly143, Thr26, and Glu189. Compound **13** occupied the four pockets of the M^pro^ due to the presence of a long ω-fatty acid chain (Figure 11). For the binding mode of compound **13** against spike glycoproteins, it formed one hydrogen bond with Tyr28 and two hydrophobic interactions with Tyr269 (Figure 12). Finally, the binding mode of compound **13** against the COVID-19 nucleocapsid phosphoprotein showed one hydrogen bond with Thr76. In addition, it formed one hydrophobic interaction with Trp52 (Figure 13). On the other hand, compound **7** exhibited good affinity into the active site of the COVID-19 membrane glycoprotein showing one hydrogen bond with Asp266. In addition, it formed four hydrophobic interactions with His65, Pro265, Val552, and Asp266 (Figure 14).

### 3.2. Correlation of c Log P with Free energy of Binding against SARS-CoV-2 Target Proteins

**Figure 15:**
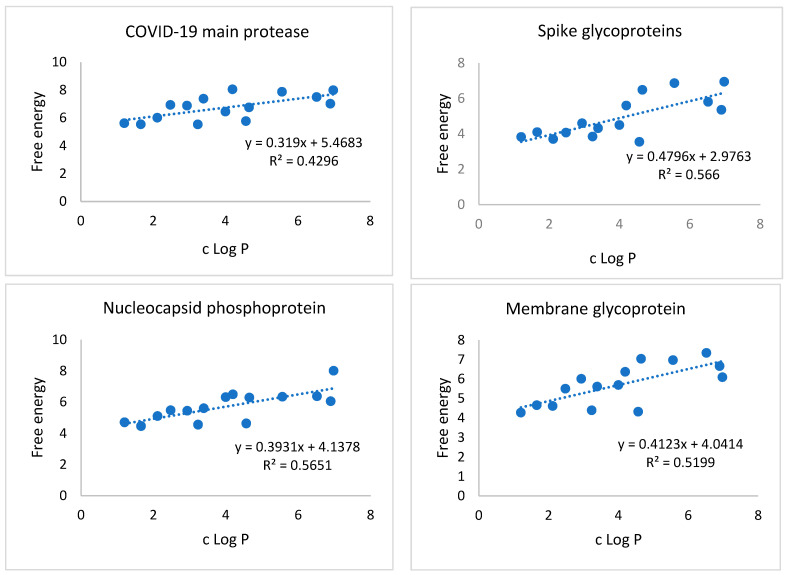
Correlation of c Log P with the free energy of binding against SARS-CoV-2 target proteins.

The calculated partition coefficients (c Log P) of the examined compounds were calculated using Discovery Studio 4.0 and summarized in Table 1. Then we investigated the correlation between c Log P and free energy of binding against SARS-CoV-2 target proteins. Simple linear regression analysis was carried out to plot the values of c Log P against the corresponding free energy of binding. The obtained coefficients of determination (R^2^) indicated that there were moderate correlations between c Log P and the free energy of binding against SARS-CoV-2 spike glycoproteins, nucleocapsid phosphoprotein, and membrane glycoprotein with R^2^ values of 0.566, 0.5651, and 0.5199, respectively (Figure 15). For the COVID-19 main protease, there were mild correlations between the c Log P and the free energy of binding with R^2^ value of 0.4296. On the other hand, there was no correlation between the c Log P and the free energy of binding against nsp10 (R^2^ = 0.1116).

### 3.3. Structure-Activity Relationship (SAR) 

Firstly, the effect of the length of the side chain on binding affinity has been explored. The decreased binding affinity of compounds **1**, **2**, **3**, **4**, **8**, and **15** (incorporating short alkyl side chain) than the corresponding members incorporating long alky side chain (**5**, **6**, **7**, **9**, **10**, **11**, **12**, and **14**) indicated that the long chain group is more preferred computationally than the shorter chain against the target receptors. In addition, it was found that the side chains with more hydrophilic characters (e.g., compounds **4** and **13**) were more effective than those with less hydrophilic groups (e.g., compounds **3** and **14**). We then investigated the impact of the main nucleus on the binding affinity of the examined compounds. Depending on the binding modes of compounds **5** and **13** (incorporating bulky nuclei), it indicated that increasing of the main nucleus bulkiness leads to an increase in the binding affinity.

### 3.4. In Silico ADMET Analysis

**Figure 16:**
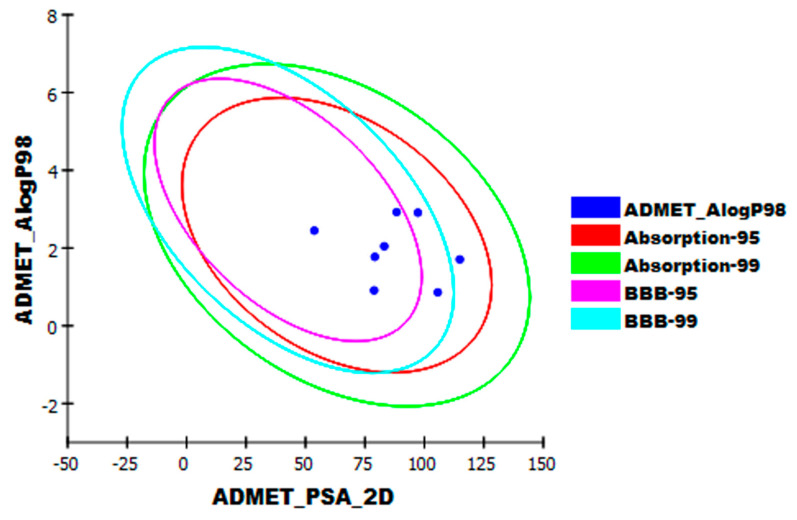
The expected ADMET study.

The promising results of these docking studies enabled us to explore the ADMET characteristics and toxicity properties of the examined alkaloids. ADMET experiments can predict different properties of these chemicals including their oral absorption, bioavailability, the ability to penetrate the blood brain barrier (BBB), their distribution, and their excretion. These properties offer valuable information about possible dose, route of administration, and the safety of the examined drugs. Furthermore, these data help to reduce the risk of a compound’s late-stage attrition. ADMET studies were carried out for 15 guanidine alkaloids. Daclatasvir (well-studied as an antiviral) was used as a reference drug. ADMET studies include many descriptors. (i) blood brain barrier penetration, which predicts blood brain barrier penetration of a molecule; (ii) intestinal absorption, which predicts human intestinal absorption (HIA) after oral administration; (iii) aqueous solubility, which predicts the solubility of each compound in the water at 25 °C; (iv) CYP2D6 binding, which predicts cytochrome P450 2D6 enzyme inhibition; (v) hepatotoxicity, which predicts the potential hepatotoxicity of a given compound; and (vi) plasma protein binding, which predicts the fraction of drug that while be bound by plasma proteins [81]. Discovery Studio 4.0 was used to predict ADMET descriptors for all compounds. The predicted descriptors are listed in Table 2. The results revealed that the tested compounds have low or very low BBB penetration levels except for compounds, monalidine A (**8**) and crambescidin 359 (**15**), which showed high levels of BBB penetration. Accordingly, it might be suggested that such compounds were expected to be safe to the Central Nervous System (CNS). The bicyclic compounds, **1** and **9**, together with the pentacyclic compounds, **5** and **6** and **12** and **13**, showed optimal range levels of ADMET aqueous solubility. Intestinal absorption is the percentage of a drug that is absorbed across the gut wall [82]. A well-absorbed drug is one that is absorbed at least 90% into the human bloodstream [83]. According to in silico ADMET studies, the bicyclic compounds **1**, **2**, **3**, and **8**, together with the pentacyclic compound **15** were predicted to have good intestinal absorption levels, while compounds **4**, **7**, **9**, **10**, and **14** showed moderate absorption levels. The cytochrome P450 2D6 (CYP2D6) model predicts the potential of a compound to inhibit CYP2D6 enzyme using 2D chemical structure as input. CYP2D6 is an essential enzyme involved in the metabolism of a wide range of substrates in the liver. Therefore, CYP2D6 inhibition is needed as part of the regulatory procedures in the drug discovery process [84]. All examined members were predicted to be non-inhibitors of CYP2D6 except monalidine A (**8**). Hepatotoxicity prediction of such compounds revealed that all compounds are non-hepatotoxic except compound monalidine A (**8**). Consequently, the liver dysfunction side effect is not expected upon administration of these compounds. The plasma protein binding model predicts whether a compound is likely to be highly bound (≥90% bound) to carrier proteins in the blood [85]. All compounds were expected to bind plasma protein less than 90% except compound **8** (Figure 16).

### 3.5. Toxicity Studies 

A toxicity prediction was carried out for the 15 guanidine alkaloids based on validated models in Discovery Studio software [86,87] as follows: (i) FDA rodent carcinogenicity which computes the probability of a chemical being a carcinogen; (ii) carcinogenic potency TD_50_ which predicts the tumorigenic dose rate 50 (TD_50_) of a chemical in a rodent chronic exposure toxicity test [88]; (iii) rat maximum tolerated dose which predicts the rat maximum tolerated dose (MTD) of a chemical [89,90]; (iv) rat oral LD_50_ which predicts the rat oral acute median lethal dose (LD_50_) in the toxicity test of a chemical [91]; and (v) rat chronic lowest observed adverse effect level (LOAEL) which predicts the rat chronic LOAEL value of a chemical [92,93]. As shown in Table 3, the tested compounds showed in silico expected low toxicity against the tested models. For the FDA rodent carcinogenicity model, the tested compounds were expected to be non-carcinogenic. For the carcinogenic potency TD_50_ mouse model, all compounds showed TD_50_ values higher than that of the reference drug, Daclatasvir. Regarding the rat maximum tolerated dose model, the compounds showed maximum tolerated doses with a range of 0.027–0.350 g/kg body weight, which are all higher than Daclatasvir (0.022 g/kg body weight). For the rat oral LD_50_ model, compounds **4**–**15** showed oral LD_50_ values ranging from 1.829 to 13.415 mg/kg body weight/day. These values are higher than that of Daclatasvir (0.677 mg/kg body weight/day). For the rat chronic LOAEL model, compounds **1**–**4** and **8**–**11** showed LOAEL values ranging from 0.0165 to 0.0450 g/kg body weight. These values are similar or higher than that of Daclatasvir (0.0063 g/kg body weight). Compounds **5**–**7** and **12**–**15** showed LOAEL values of ranging from 0.0012 to 0.0019 g/kg body weight, which is less than Daclatasvir.

### 3.6. Molecular Dynamics Simulation for Compounds ***5*** and ***13***

Six MD simulations were performed to confirm the stability of the ligands binding to their biological targets over a 100 ns time frame. Compound **5** was subjected to MD with the COVID-19 main protease (PDB ID: 6lu7), nucleocapsid phosphoprotein (PDB ID: 6VYO), and nsp10 (PDB ID: 6W4H), and compound **13** was assessed on the COVID-19 main protease (PDB ID: 6lu7), spike glycoproteins (PDB 30 ID: 6VYB) and nucleocapsid phosphoprotein (PDB ID: 6VYO).

**Figure 17:**
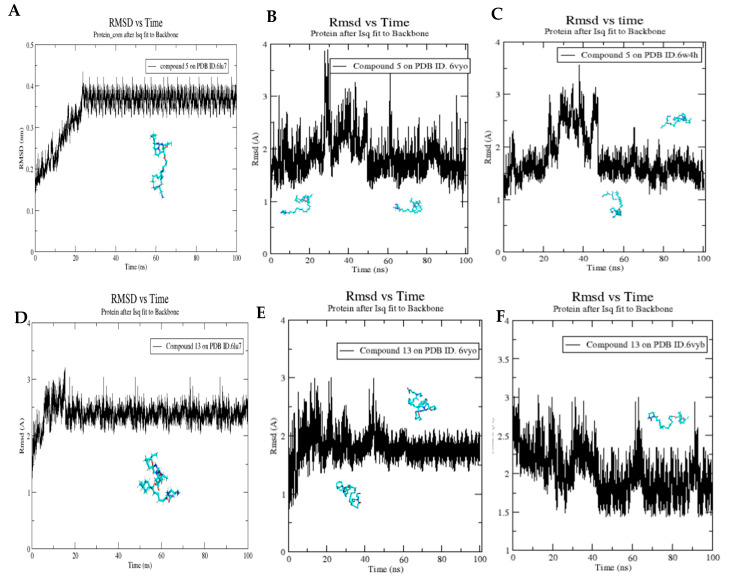
Molecular dynamics simulations of compound **5** bound to PDB ID. 6lu7 (**A**), 6VYO (**B**), and 6W4H (**C**), and compound **13** bound to PDB ID. 6lu7 (**D**), 6VYB (**E**), and 6VYO (**F**). RMSD analysis of compounds **5** and **13** binding with their biotarget proteins showing the most stable ligand conformers.

**Figure 18:**
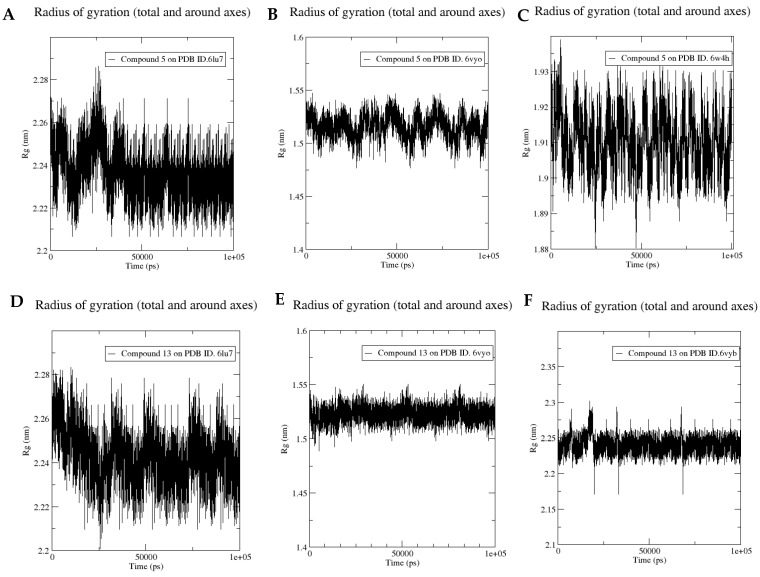
Molecular dynamics of compound **5** bound to PDB ID. 6lu7 (**A**), 6VYO (**B**) and 6W4H (**C**) and compound **13** bound to PDB ID. 6lu7 (**D**), 6VYB (**E**) and 6VYO (**F**). Radius of gyration of the protein along the simulation time.

To analyze the stability of the simulated system, the conformational changes of protein-ligand complexes were analyzed over the course of the 100 ns MD simulation using two methods: RMSD with respect to the initial structure and the radius of gyration shown in (Figure 17 and Figure 18), respectively.

Compound **5** reached a stable conformation after 30 ns on the COVID-19 main protease active site (Figure 17A) with RMSD fluctuating around 3.5 Å and radius of gyration fluctuating around 2.24 nm (Figure 18A). When it binds to nucleocapsid phosphoprotein, compound **5** shows two stable conformations; one at the first 20 ns of the simulation and the other after 50 ns from the beginning of the simulation with average RMSD of 1.5 Å (Figure 17B) and its radius of gyration fluctuates between 1.5 and 1.55 nm (Figure 18B) indicating its relative stability throughout the last 50 ns of the simulation. Similar results have been obtained when it binds to nsp10, RMSD reaches stability after 50 ns of the simulation with average RMSD 1.5 Å (Figure 17C) and the radius of gyration is fluctuating between 1.88 and 1.93 nm (Figure 18C).

Regarding compound **13**, it shows very stable binding with the COVID-19 main protease with a very stable conformation after only 20 ns with an average RMSD of 2.3 Å (Figure 17D) and a radius of gyration fluctuating between 2.22 and 2.28 nm (Figure 18D). Furthermore, it reached a stable binding conformation with the nucleocapsid phosphoprotein active site after 50 ns with RMSD fluctuating around 2 Å, indicating its relative stability throughout the last 50 ns of the simulation (Figure 17E). Regarding the radius of gyration, it fluctuates between 1.5 and 1.55 nm throughout the whole simulation showing only minor fluctuations which further confirms the stability of the simulations (Figure 18E). Finally, compound 15’s complex with spike glycoprotein reaches two stable structures after 40 ns with a RMSD fluctuating between 1.5 and 2 Å (Figure 17F) and with very small fluctuations of radius of gyration ranging between 2.2 and 2.25 nm (Figure 18F).

**Figure 19:**
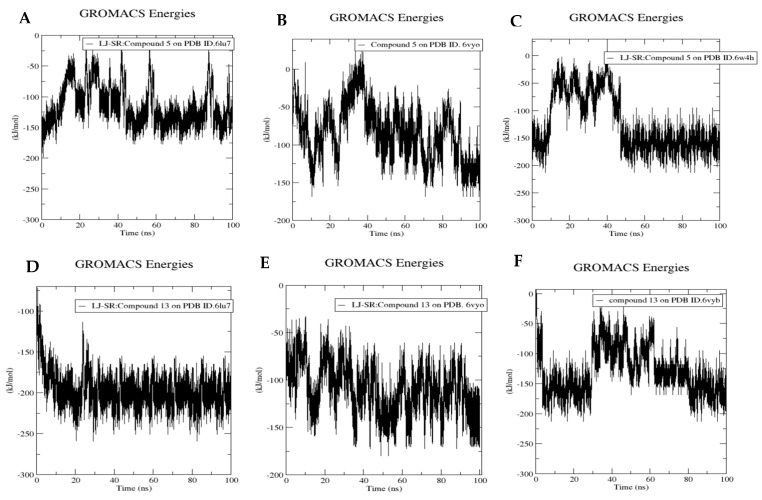
Molecular dynamics of compound **5** bound to PDB ID. 6lu7 (**A**), 6VYO (**B**) and 6W4H (**C**) and compound **13** bound to PDB ID. 6lu7 (**D**), 6VYB (**E**) and 6VYO (**F**). Binding energy using LJ-SR.

Protein-ligand interactions were calculated to quantify the strength of the interaction between each ligand and its biotarget through computing the energies of the nonbonded interactions. Compound **5** showed very low energy with all the targets; −150 kJ/mol with the COVID-19 main protease, −150 kJ/mol with nucleocapsid phosphoprotein and −200 kJ/mol with nsp10 (Figure 19A–C). Similarly, compound **13** shows very low binding energy: −200 kJ/mol with the COVID-19 main protease, −150 kJ/mol with nucleocapsid phosphoprotein and −200 kJ/mol with spike glycoproteins (Figure 19D–F). These results further confirm the stability of the formed protein-ligand complexes.

## 4. Conclusions

Fifteen structurally divergent polycyclic guanidine alkaloids were comprehensively investigated for their virtual antiviral potentials against five SARS-Cov-2 (COVID-19) proteins. The pentacyclic guanidinic scaffolds, crambescidins 786 (**5**) and 826 (**13**) displayed the best binding modes and free energies among the 15 investigated compounds. These docking results were confirmed with the very stable molecular dynamics simulation, opening the door to conduct more research (in vitro and in vivo) on compounds **5** and **13** as expected anti COVID-19 candidates. Compounds **5** and **13** exhibited very promising in silico ADMET results as well as showing high safety margins against five toxicity models. The promising ADMET and toxicity results increase the likelihood of compounds **5** and **13** being used as drugs.

## Figures and Tables

**Table 1:** Free energies of binding for fifteen marine guanidine alkaloids (**1**–**15**) to SARS-CoV-2 target proteins, and their calculated partition coefficient (c log P).

Compound	COVID-19 Main Protease	Spike Glycoproteins	Nucleocapsid Phosphoprotein	Membrane Glycoprotein	nsp10	c Log P
Monanchoradin A (**1**)	−5.62	−3.83	−4.70	−4.27	−6.12	1.204
Monanchoradin B (**2**)	−5.54	−4.10	−4.46	−4.65	−5.73	1.66
Monanchoradin C (**3**)	−6.01	−3.71	−5.10	−4.61	−6.08	2.116
Dehydrocrambescin A2 418 (**4**)	−6.45	−4.50	−6.31	−5.69	−7.19	3.998
Crambescidin 786 (**5**)	−8.05	−5.60	−6.49	−6.37	−9.06	4.195
Crambescidin 814 (**6**)	−7.87	−6.87	−6.34	−6.97	−7.50	5.563
Norcrambescidic acid (**7**)	−7.50	−5.81	−6.37	−7.34	−7.35	6.52
Monalidine A (**8**)	−5.77	−3.55	−4.63	−4.32	−5.63	4.566
(−)-crambescin A2 392 (**9**)	−6.93	−4.07	−5.47	−5.50	−6.61	2.479
(−)-crambescin A2 406 (**10**)	−6.88	−4.60	−5.44	−6.01	−10.54	2.936
(−)-crambescin A2 420 (**11**)	−7.38	−4.32	−5.60	−5.61	−6.53	3.392
Crambescidin 800 (**12**)	−6.75	−6.49	−6.29	−7.04	−7.22	4.651
Crambescidin 826 (**13**)	−7.99	−6.95	−8.01	−6.09	−8.39	6.979
Crambescidic acid (**14**)	−7.02	−5.36	−6.05	−6.66	−7.38	6.898
Crambescidin 359 (1**5**)	−5.53	−3.85	−4.55	−4.39	−4.72	3.235
Co-crystallized ligand (PRD_002214)	−8.18	-	-	-	-	-
Co-crystallized ligand (NAG)	-	−3.56	-	-	-	-
Co-crystallized ligand (MES)	-	-	−3.80	-	-	-
Co-crystallized ligand (NAG)	-	-	-	−3.63	-	-
Co-crystallized ligand (SAM)	-	-	-	-	−5.77	-

**Table 2:** Predicted ADMET for 15 guanidine alkaloids and reference drug, Daclatasvir.

Compounds	BBB Level ^a^	Solubility Level ^b^	Absorption Level ^c^	CYP2D6 Prediction ^d^	Hepatotoxicity Prediction ^e^	PPB Prediction ^f^
Monanchoradin A (**1**)	3	4	0	FALSE	FALSE	FALSE
Monanchoradin B (**2**)	3	3	0	FALSE	FALSE	FALSE
Monanchoradin C (**3**)	3	3	0	FALSE	FALSE	FALSE
Dehydrocrambescin A2 418 (**4**)	4	3	2	FALSE	FALSE	FALSE
Crambescidin 786 (**5**)	4	4	3	FALSE	FALSE	FALSE
Crambescidin 814 (**6**)	4	4	3	FALSE	FALSE	FALSE
Norcrambescidic acid (**7**)	4	2	2	FALSE	FALSE	FALSE
Monalidine A (**8**)	1	2	0	TRUE	TRUE	TRUE
(-)-crambescin A2 392 (**9**)	4	4	1	FALSE	FALSE	FALSE
(-)-crambescin A2 406 (**10**)	4	3	1	FALSE	FALSE	FALSE
(-)-crambescin A2 420 (**11**)	4	3	2	FALSE	FALSE	FALSE
Crambescidin 800 (**12**)	4	4	3	FALSE	FALSE	FALSE
Crambescidin 826 (**13**)	4	4	3	FALSE	FALSE	FALSE
Crambescidic acid (**14**)	4	2	2	FALSE	FALSE	FALSE
Crambescidin 359 (**15**)	1	2	0	FALSE	FALSE	FALSE
Daclatasvir	4	3	3	FALSE	TRUE	TRUE

^a^ BBB level, blood brain barrier level, 0 = very high, 1 = high, 2 = medium, 3 = low, 4 = very low. ^b^ Solubility level, 1 = very low, 2 = low, 3 = good, 4 = optimal. ^c^ Absorption level, 0 = good, 1 = moderate, 2 = poor, 3 = very poor. ^d^ CYP2D6, cytochrome P2D6, TRUE = inhibitor, FALSE = non inhibitor. ^e^ Hepatotoxicity, TRUE = hepatotoxic, FALSE = non-hepatotoxic. ^f^ PBB, plasma protein binding, FALSE means less than 90%, TRUE means more than 90%.

**Table 3:** Toxicity properties of the most promising compounds (**1**–**15**).

Compounds	FDA Rodent Carcinogenicity	Carcinogenic Potency TD_50_ Mouse ^a^	Rat Maximum Tolerated Dose (Feed) ^b^	Rat Oral LD_50_^b^	Rat Chronic LOAEL ^b,^*
Monanchoradin A (**1**)	Non-Carcinogen	51.0661	0.085	0.399	0.0168
Monanchoradin B (**2**)	Non-Carcinogen	52.712	0.091	0.457	0.0167
Monanchoradin C (**3**)	Non-Carcinogen	54.2866	0.098	0.509	0.0166
Dehydrocrambescin A2 418 (**4**)	Non-Carcinogen	19.5925	0.573	10.139	0.0450
Crambescidin 786 (**5**)	Non-Carcinogen	1.91771	0.063	10.559	0.0019
Crambescidin 814 (**6**)	Non-Carcinogen	1.91977	0.071	13.415	0.0017
Norcrambescidic acid (**7**)	Non-Carcinogen	5.77105	0.043	11.836	0.0013
Monalidine A (**8**)	Non-Carcinogen	32.2161	0.123	3.156	0.0448
(-)-crambescin A2 392 (**9**)	Non-Carcinogen	39.9613	0.310	2.634	0.0171
(-)-crambescin A2 406 (**10**)	Non-Carcinogen	40.6645	0.329	2.970	0.0168
(-)-crambescin A2 420 (**11**)	Non-Carcinogen	41.3406	0.350	3.269	0.0165
Crambescidin 800 (**12**)	Non-Carcinogen	1.91899	0.065	11.440	0.0018
Crambescidin 826 (**13**)	Non-Carcinogen	1.30045	0.042	14.200	0.0012
Crambescidic acid (**14**)	Non-Carcinogen	5.07065	0.040	8.153	0.0018
Crambescidin 359 (**15**)	Non-Carcinogen	0.779067	0.027	1.829	0.0021
Daclatasvir	Non-Carcinogen	0.970599	0.022	0.677	0.0063

^a^ mg/kg body weight/day, ^b^ Unit: g/kg body weight, * Lowest-observed-adverse-effect level (LOAEL).

## Data Availability

Not applicable.
